# Physician Perceptions on Colonoscopy Quality: Results of a National Survey of Gastroenterologists

**DOI:** 10.1155/2014/510494

**Published:** 2014-03-06

**Authors:** Ziad F. Gellad, Corrine I. Voils, Li Lin, Dawn Provenzale

**Affiliations:** ^1^Center for Health Services Research in Primary Care, Durham Veterans Affairs Medical Center, Durham, NC 27710, USA; ^2^Department of Medicine, Duke University Medical Center, Durham, NC 27705, USA; ^3^Duke Clinical Research Institute, Duke University Medical Center, Durham, NC 27705, USA; ^4^VA Cooperative Studies Epidemiology Center, Durham, NC 27710, USA

## Abstract

*Background*. Quality indicators for colonoscopy have been developed, but the uptake of these metrics into practice is uncertain. Our aims were to assess physician perceptions regarding colonoscopy quality measurement and to quantify the perceived impact of quality measurement on clinical practice. *Methods*. We conducted in-person interviews with 15 gastroenterologists about their perceptions regarding colonoscopy quality. Results from these interviews informed the development of a 34-question web-based survey that was emailed to 1,500 randomlyselected members of the American College of Gastroenterology. *Results*. 160 invitations were undeliverable, and 167 out of 1340 invited physicians (12.5%) participated in the survey. Respondents and nonrespondents did not differ in age, sex, practice setting, or years since training. 38.8% of respondents receive feedback on their colonoscopy quality. The majority of respondents agreed with the use of completion rate (90%) and adenoma detection rate (83%) as quality indicators but there was less enthusiasm for withdrawal time (61%). 24% of respondents reported usually or always removing diminutive polyps solely to increase their adenoma detection rate, and 20% reported prolonging their procedure time to meet withdrawal time standards. *Conclusions*. A minority of respondents receives feedback on the quality of their colonoscopy. Interventions to increase continuous quality improvement in colonoscopy screening are needed.

## 1. Introduction

The use of colonoscopy for colorectal cancer (CRC) screening is increasing in frequency [1–3] and has become the dominant screening modality in the United States Medicare population [4]. Numerous observational studies have demonstrated the preventive benefits of colonoscopy [5–10], and several large randomized clinical trials are underway. However, colonoscopy is not a perfect test; population-based studies suggest that colonoscopy can miss cancerous lesions in 2%–6% of exams [11–14]. Furthermore, recent studies have underscored the limitation of colonoscopy in preventing CRC, especially on the right side of the colon [8, 15, 16].

While there may be biologic explanations for this suboptimal impact, studies have found that endoscopist behavior also plays a key role [17–19]. In fact, there is well established variation among endoscopists in colonoscopy surveillance recommendations [20, 21] and adenoma and polyp detection rates [22–24]. Furthermore, low detection rates have been associated with interval cancer risk [25]. Quality metrics for colonoscopy have been proposed by the national gastroenterology societies [26] and advocated in high-profile settings [27], but the dissemination and use of these metrics in practice is uncertain. Furthermore, few studies have attempted to understand the reasons underlying this variation in quality metrics.

To address these uncertainties, we undertook a survey of gastroenterologists to better understand the dissemination of best practices in colonoscopy and to assess physician perceptions regarding colonoscopy quality and quality measurement. Our hypothesis was twofold: firstly, we anticipated that, as has been observed in other fields [28], the implementation of quality measurement in colonoscopy practice, as indicated by the proportion of endoscopists who receive feedback, would be low; secondly, we believed that the perceived impact of quality measurement on clinical practice would vary widely among physicians.

## 2. Methods

### 2.1. Study Overview

The survey methodology, including a copy of the final survey instrument, has been reported [29] and is summarized below. The Duke University Medical Center Institutional Review Board approved the study and the consent procedures. Written informed consent was obtained from participating physicians for the qualitative interviews. Waiver of written informed consent for the web survey was approved by the Duke Institutional Review Board based on three factors: first, the research presented no more than minimal risk to subjects; second, participants were informed in writing of the purpose of the survey and potential risks prior to participating and had the opportunity to opt out of the survey at any point; third, the study involved no procedures for which written consent is normally required outside of the research context.

### 2.2. Qualitative Interviews

We conducted in-person interviews with 15 gastroenterologists about their perceptions regarding colonoscopy quality and quality measurement. Physicians were eligible if they were actively practicing gastroenterologists in Durham, Chapel Hill, or Raleigh, North Carolina. Electronic and mailed invitations were sent to eligible physicians. Respondents were offered a meal and a $50 cash incentive for participation. Because responses may differ according to practice setting and experience, maximum variation sampling was used, with the goal of obtaining physicians both from academic and community practice as well as those with fewer and greater than five years in practice.

Interviews occurred between October 2010 and December 2010 in a private setting. Interviews were conducted by a single nonphysician investigator (C. I. Voils) skilled in qualitative methodology. The interviewer used a structured interview guide to probe physicians about clinical practice patterns and beliefs regarding colonoscopy quality. Interviews were digitally audiorecorded. Data analysis and data collection occurred iteratively; after each interview was conducted, two team members (Z. F. Gellad and C. I. Voils) listened to the interview and compared responses to previous interviews to identify issues deserving more attention in future interviews and to determine when thematic saturation was reached.

Interviews were transcribed for analysis. Directed content analysis was used to identify important themes and concepts regarding colonoscopy quality [30]. Two team members (Z. F. Gellad, C. I. Voils) generated descriptive labels to describe themes; the coding scheme was finalized through discussion, and then the final coding scheme was applied to all transcripts by one team member (Z. F. Gellad). Responses were stratified by respondent practice type (academic versus community) and years in practice (≤5 years versus >5 years).

### 2.3. Survey Design

After reviewing the results of the qualitative work discussed above, we composed a 34-question web-based closed survey instrument, which was administered using REDCap (Research Electronic Data Capture) tools hosted at Duke University [31]. The survey was pretested and revised using cognitive interviewing with five volunteer gastroenterologists varying in age and years in practice to assure clarity [32]. Although the survey queried a variety of factors related to endoscopy practice, only results regarding colonoscopy practice and performance quality are reported herein.

### 2.4. Survey Distribution and Data Collection

Active full members of the American College of Gastroenterology (ACG) were eligible to participate (*n* = 9, 154). Members were excluded if they were Duke physicians (*n* = 14) or did not have an email address available in the database (*n* = 2579). The database was used with permission from the ACG.

We distributed the survey in two phases. In the first phase, we invited 500 randomly selected ACG members to participate in July 2011. Physicians received an introductory email from a dedicated study email account with a duke.edu domain, signed by all of the investigators, with an invitation to participate in the survey. This email contained a unique link to the online survey. Respondents were able to opt out of the study by clicking in an embedded URL link that would terminate any further contact. An email reminder was sent to all nonresponders at one week and three weeks after the introductory email. The survey was closed four weeks after the introductory email. As an incentive for participation, respondents were entered into a drawing for one of two Apple iPad2s.

Because the response rate to this first phase was less than we had foreseen, we randomly selected another 1000 physicians from the same population to participate. These ACG members were invited in the same fashion as described above in September 2011 and are included together in the analysis that follows.

### 2.5. Statistical Analysis

Physicians' characteristics are described using frequencies and percentages or medians with interquartile ranges, as appropriate. To examine the association between physician characteristics and responses to the survey questions, we performed Wilcoxon rank sum tests (for continuous variables) or *χ* ^2^ test (for categorical variables) for bivariate comparisons of physician responses by respondent sex, median years in practice, gastroenterology board certification, practice setting (academic, private/mixed), weekly colonoscopy volume (<10, 10–20, 21–30, 31–40, and >40), productivity bonus, and receipt of feedback on colonoscopy quality. We then performed multivariable analyses with the abovementioned covariates using logistic regression for items with dichotomous response options and general linear regression modeling for items with ordinal scaled polytomous responses. Items with ordinal polytomous responses were coded with higher scores corresponding to greater agreement or frequency. We used variance inflation factors to quantify collinearity among the predictor variables and removed age from the models because it showed severe redundancy with years since training. To facilitate interpretation of the model results, we reported the effect size for each covariate, namely, the proportion of total variation accounted for by the effect (*η* ^2^) in general linear models and odds ratios in the logistic regressions.

We used SAS version 9.2 (SAS Institute, Inc., Cary, North Carolina) for all analyses and considered a 2-tailed *α* level of 0.05 to be significant without adjusting for multiple comparisons.

### 2.6. Nonresponse Analysis

We retrieved demographic and training information from the American Medical Association (AMA) Physician Masterfile for 1,251 of the 1,340 physicians who received an invitation to participate in the survey. Physicians were matched to Masterfile data by name, address, email address, and National Provider Identifier. We compared respondents and nonrespondents by sex, practice setting, gastroenterology board certification, and years since most recent training using the *χ* ^2^ test for categorical variables and the Wilcoxon rank sum test for continuous variables. Due to the possibility that nonclinicians would be represented in the nonrespondent category group, gastroenterology board certification was compared only in those classified by the AMA as gastroenterologists, hepatologists, internists, and colorectal surgeons.

## 3. Results

### 3.1. Response Rate & Physician Demographics

Of the 1500 email survey invitations, 160 were returned as undeliverable, resulting in an invited population of 1340 physicians. 167 physicians responded to the survey, resulting in a participation rate of 12.5%. The demographic characteristics of survey respondents are shown in Table 1. The majority of respondents were male (87.2%), board certified (91.3%), and in private practice (67.3%). Female respondents were more likely to be employed in academic settings (60.0% versus 18.7%, *P* < 0.01) and were younger (median of 4.5 years in practice versus 20.0 years, *P* < 0.01) than male respondents.

### 3.2. Nonresponse Analysis

Responders and nonresponders did not differ significantly in age (*P* = 0.17), sex (*P* = 0.89), practice setting (*P* = 0.71), or years in practice (*P* = 0.21). There were more specialists in the nonresponder group that would not be expected to obtain GI board certification (data not shown). When these specialists were excluded from the sample and only colorectal surgeons, hepatologists, gastroenterologists, and internists were included, the respondents and nonrespondents did not significantly differ in terms of board certification in gastroenterology (91.3% versus 87.5%, *P* = 0.19).

### 3.3. Receipt of Quality Feedback

38.8% of respondents reported receiving feedback on the quality of their colonoscopy. There were no significant differences in receipt of feedback by endoscopist sex, years in practice, board certification, practice setting, receipt of a productivity bonus, or colonoscopy volume (Table 2). As shown in Table 3, the odds of discussing missed lesions were greater among respondents who received feedback than those that did not receive feedback (OR 4.05, 95% CI 1.53–10.8), independent of other factors. The odds of retroflexing in the right colon were also greater among respondents who received feedback (*P* < 0.01), although the effect size was small (Table 4). There was no association between receipt of feedback and attempt to intubate the terminal ileum (*P* = 0.65), retroflexion in the rectum (*P* = 0.72), prolongation of the procedure to meet quality standards for withdrawal time (*P* = 0.85), or removal of polyps solely to increase adenoma detection rate (*P* = 0.87).

### 3.4. Physician Perceptions Regarding Colonoscopy Quality Measurement

**Figure 1:**
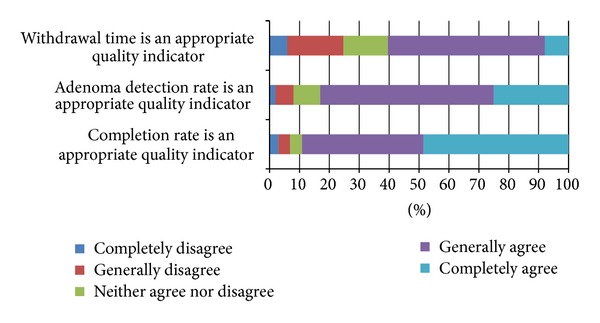
Physician perceptions about colonoscopy quality measures.

Figure 1 presents survey results of physician perceptions regarding colonoscopy quality measures. The majority of respondents agreed with the use of completion rate (90%) and adenoma detection rate (83%) as quality indicators. Although they are still a majority, fewer respondents supported the use of withdrawal time (61%) as an appropriate quality indicator. Respondents felt that the benchmark rates for withdrawal time and adenoma detection were too low (24% and 25%), about right (69% and 66%), or too high (8% and 9%). Respondents who receive feedback on the quality of their colonoscopy were more likely to say recommended detection rates were too low, even after adjusting for other factors (*P* < 0.01) (Table 5). In contrast, increasing years in practice were significantly associated with answering that the recommended rates were too high (*P* < 0.01). In regard to a benchmark withdrawal time of 6 minutes, responses did not significantly differ among measured covariates.

### 3.5. Colonoscopy Practice Variation: Detection and Removal

**Figure 2:**
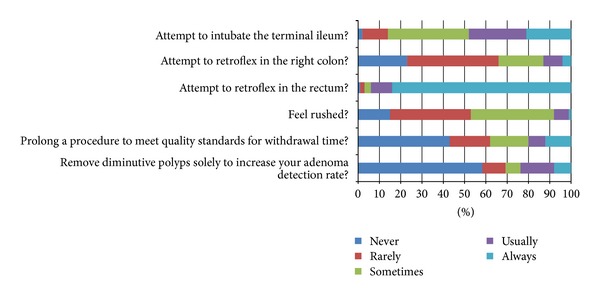
While performing screening and surveillance colonoscopies, how often do you…?

Figure 2 displays the variation in colonoscopy practice among respondents. While the majority of respondents (94%) reported usually or always attempting to retroflex in the rectum, only 13% reported usually or always attempting to retroflex in the right colon and 48% to intubate the terminal ileum. In multivariable analysis (Table 4), receipt of quality feedback (*P* < 0.01) and male sex (*P* = 0.05) were significantly associated with retroflexion in the right colon. Also, twice as many respondents in academic practice reported usually or always attempting retroflexion in the right colon as compared to private/mixed practice (22.3% versus 11.3%, *P* < 0.01). 24% of respondents reported usually or always removing diminutive polyps solely to increase their adenoma detection rate. Similarly, 20% of respondents reported usually or always prolonging their procedure to meet quality standards for withdrawal time. None of the examined covariates was associated with respondents' responses to these questions (Table 4).

### 3.6. Colonoscopy Practice Variation: Surveillance

**Figure 3:**
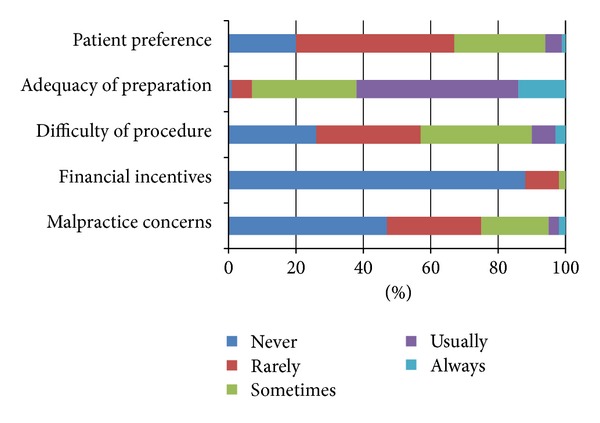
Please rate how often do you shorten a surveillance recommendation compared to the guidelines based on the following factors…?

The survey also asked respondents to rate factors that result in shortened surveillance recommendations (Figure 3). While financial incentives did not appear to be a major driver of behavior, difficulty of procedure, adequacy of preparation, and, to a lesser extent, patient preference and malpractice concerns did play a role. For example, 62% of respondents reported usually or always shortening a surveillance recommendation because of a suboptimal preparation. 10% of respondents reported shortening a surveillance recommendation because of a difficult procedure. While only 6% of respondents reporting usually or always shortening surveillance intervals based on patient preference, respondents in private practice were significantly more likely, than those in academic practice, to do so in multivariable analysis (*P* < 0.01) (Table 6). Respondents in private practice were also more likely to shorten surveillance intervals based on malpractice concerns (*P* < 0.01) as were the respondents with fewer years in practice (*P* < 0.01) and lower colonoscopy volume (*P* = 0.04), although effect sizes were small. Surveillance patterns in response to adequacy of the colon prep varied only by sex, with 85.7% of females reporting usually or always shortening a surveillance recommendation based on inadequacy of the preparation as compared to 58.9% of men (*P* < 0.01).

## 4. Discussion

In this national sample of gastroenterologists, we detected a number of noteworthy findings. For one, the majority of respondents do not yet receive feedback on the quality of their colonoscopy, even a decade after quality measures were first proposed [33]. Nonetheless, the majority of respondents support the use of completion rate (90%) and adenoma detection rate (83%) for quality improvement. There was significantly less support for withdrawal time as a quality measure, perhaps in part related to the controversy regarding its clinical significance [33].

The slow dissemination and implementation of quality measurement in practice is not surprising given the experience in other fields [28]. Our survey instrument did not specifically explore reasons for lack of feedback, but we speculate that a number of factors play an important role, including the uncertainty regarding the eventual use of these measures by external forces and the logistical and technical difficulties in collecting necessary data [34–36]. These challenges are not new to quality measurement [37] nor are they unique to gastroenterology [38]. Nonetheless, they represent important issues to address in advancing the implementation of continuous quality improvement into gastroenterology practice. Indeed, practices that embrace quality measurement will be better positioned to demonstrate their value to payers and patients [39] and will assure they are providing the best care available to their patients [40].

Another notable finding from our survey is the acknowledgment of the part of practicing physicians of behavior change as a response to colonoscopy quality measures. For example, a minority of respondents acknowledged that they may prolong procedures to meet withdrawal time standards and remove diminutive polyps solely to increase their adenoma detection rate. The impact of these behaviors on patient outcome is unclear and underscores the difficulty in relying on process measures rather than outcomes for quality assessment [41].

Finally, survey respondents identified a number of factors that may lead them to shorten surveillance intervals compared to recommendations, including inadequacy of bowel preparation, malpractice concerns, difficulty of the procedure, and patient preferences. While other studies have extrapolated potential reasons for nonadherence with surveillance guidelines from physician survey data [21, 42], our analysis is unique in that it first identified potential factors from qualitative interviews and then quantified through the survey the extent to which those factors exist among physicians. Adequacy of preparation had the strongest impact and is a known factor affecting surveillance recommendations. Our results also confirm the speculation that physicians may sometimes shorten surveillance intervals because of concerns regarding malpractice; respondents in private practice and those with fewer years in practice were significantly more likely to do so when compared to their colleagues. Patient preference and the difficulty of the procedure also contributed to shortened surveillance intervals. While the contribution of each of these individual factors may be small, the collective impact may help explain some of observed “overuse” of surveillance colonoscopy [20, 43].

This study had some limitations. Most notable is the low participation rate (12.5%). Although this rate is lower than that we had anticipated in designing the survey, it falls within the range reported by other GI society-based surveys (9.5%–29%) [44–46]. Furthermore, nonresponse bias is a bigger threat to the validity of findings than a low response rate per se [47, 48]. In support of our findings, we found no evidence of such bias in our analysis. Another limitation is that we did not have sufficient numbers of nongastroenterology physicians in our survey population to make inferences regarding other physicians performing colonoscopy. Finally, despite the lack of detectable nonresponse bias, we cannot exclude selection bias if members of the American College of Gastroenterology systematically differ from nonmembers.

In summary, in a survey of practicing gastroenterologists, we found that a minority of physicians reported receiving feedback on the quality of their colonoscopy and that the majority of respondents support currently available colonoscopy quality process measures. In addition, we found evidence that quality metrics have led to changes in physician practice, both in academic and community settings. Studies assessing reasons for lack of performance feedback and the best mechanism for feedback will be helpful in identifying opportunities for interventions to increase the implementation of continuous quality improvement in practice.

## Figures and Tables

**Table 1:** Characteristics of physician respondents & nonrespondents.

Characteristic	Respondents^a^	Nonrespondents^a^	*P* value
*AMA data *			
Age, median (25th, 75th percentile)	52.0 (42.5, 59.0)	50.0 (41.0, 58.0)	0.17
Sex			
Male	136 (87.2%)	925 (86.8%)	0.89
Female	20 (12.8%)	141 (13.2%)	
Years in practice			
≤5 years	28 (18.5%)	212 (20.4%)	0.17
>5 years	123 (81.5%)	825 (79.6%)	
Gastroenterology board certification^b^			
Yes	136 (91.3%)	862 (87.5%)	0.19
No	13 (8.72%)	123 (12.5%)	
Practice setting^c^			
Solo practice	21 (12.6%)	145 (12.4%)	0.59
Group practice	97 (58.1%)	635 (54.1%)	
Employed	20 (12.0%)	135 (11.5%)	
Other/missing	29 (17.4%)	258 (22.0%)	
*Survey data *			
Practice setting			
Academic	38 (23.0%)		
Private	111 (67.3%)	n/a	n/a
Mixed	16 (9.7%)		
Average number of colonoscopies/week			
<10	19 (11.6%)	n/a	n/a
10–20	72 (43.9%)		
21–30	51 (31.1%)		
31–40	16 (9.8%)		
>40	6 (3.7%)		
Proportion of practice made up of colonoscopy			
<25%	33 (20.0%)	n/a	n/a
25–50%	89 (53.9%)		
51–75%	36 (21.8%)		
76–100%	7 (4.2%)		
Productivity bonus?			
Yes	81 (49.7%)	n/a	n/a
No	82 (50.3%)		
Receive feedback on quality of colonoscopy			
Yes	62 (38.8%)	n/a	n/a
No	98 (61.2%)		

^a^For the nonresponse analysis, AMA Masterfile data were available for 167 respondents and 1173 nonrespondents.

^b^After including only providers who might be expected to obtain certification in this specialty (internal medicine, gastroenterology, hepatology, and colorectal surgery).

^c^Practice setting data was missing in AMA Masterfile in 28 (16.8%) of respondents and 253 (21.6%) of nonrespondents. Practice setting was categorized using AMA classifications as solo practice (self-employed solo practice), group practice (two physician practice-owner, group practice), employed (HMO, medical school, nongovernmental hospital, government hospital), or others.

**Table 2:** Factors impacting the receipt of feedback regarding colonoscopy quality in multivariable analyses^a^.

Variable	No feedback (*n* = 98)	Yes feedback (*n* = 62)	Odds ratio (95% CI)^b^	*P* value
Sex				
Male	80 (87.0%)	52 (91.2%)	—	0.92
Female	12 (13.0%)	5 (8.8%)	0.94 (0.27–3.21)	
Years in practice, mean (SD)	17.2 (12.2)	19.1 (11.4)	1.01 (0.98–1.05)^c^	0.43
Gastroenterology board certification				
Yes	79 (80.6%)	53 (85.5%)	—	0.19
No	19 (19.4%)	9 (14.5%)	0.44 (0.12–1.53)	
Practice setting				
Academic	25 (25.5%)	11 (17.7%)	0.78 (0.31–1.98)	0.60
Private/mixed	73 (74.5%)	51 (82.3%)	—	
Productivity bonus				
Yes	47 (48.0%)	32 (53.3%)	1.36 (0.67–2.75)	0.39
No	51 (52.0%)	28 (46.7%)	—	
Average number of colonoscopies/week				
<10	13 (13.3%)	5 (8.1%)	0.57 (0.15–2.15)	0.73
10–20	45 (45.9%)	26 (41.9%)	—	
21–30	28 (28.6%)	22 (35.5%)	1.45 (0.64–3.25)	
31–40	8 (8.2%)	7 (11.3%)	1.39 (0.44–4.40)	
>40	4 (4.1%)	2 (3.2%)	1.14 (0.17–7.55)	

^a^Odds ratio of receipt of feedback based on the results of multivariable logistic regression analysis.

^b^The most frequently observed category was used as the reference.

^c^Per one year increase in years in practice.

**Table 3:** Impact of quality feedback on colonoscopy practice in multivariable analyses.

Variable	No feedback (*n* = 98)	Yes feedback (*n* = 62)	*P* value
Discuss risk of missed lesions in consent			
Yes	65 (67.0%)	52 (86.7%)	<0.01
No	32 (33.0%)	8 (13.3%)	
Attempt to intubate the terminal ileum			
Never	2 (2.0%)	1 (1.6%)	0.65
Rarely	9 (9.2%)	10 (16.1%)	
Sometimes	40 (40.8%)	21 (33.9%)	
Usually	33 (33.7%)	10 (16.1%)	
Always	14 (14.3%)	20 (32.3%)	
Attempt to retroflex in the right colon			
Never	28 (28.6%)	8 (12.9%)	<0.01
Rarely	47 (48.0%)	22 (33.5%)	
Sometimes	16 (16.3%)	17 (27.4%)	
Usually	4 (4.1%)	11 (17.7%)	
Always	3 (3.1%)	4 (6.5%)	
Attempt to retroflex in the rectum			
Never	2 (2.0%)	0	0.72
Rarely	2 (2.0)	1 (1.6%)	
Sometimes	3 (3.1%)	1 (1.6%)	
Usually	9 (9.2%)	7 (11.3%)	
Always	82 (83.7%)	53 (85.5%)	
Prolong procedure to meet quality standards for withdrawal time			
Never	42 (42.9%)	26 (43.3%)	0.85
Rarely	16 (16.3%)	14 (23.3%)	
Sometimes	18 (18.4%)	11 (18.3%)	
Usually	10 (10.2%)	2 (3.3%)	
Always	12 (12.2%)	7 (11.7%)	
Remove polyps solely to increase adenoma detection rate			
Never	56 (57.1%)	39 (62.9%)	0.87
Rarely	13 (13.3%)	4 (6.5%)	
Sometimes	6 (6.1%)	5 (8.1%)	
Usually	16 (16.3%)	9 (14.5%)	
Always	7 (7.1%)	5 (8.1%)	

*P value based on multivariable linear regression model that included sex, years in practice, GI board certification, practice setting, productivity bonus, and colonoscopy volume as covariates.

**Table 4:** While performing screening and surveillance colonoscopies, how often do you…?*.

		Attempt to intubate the terminal ileum?	Attempt to retroflex in the right colon?	Attempt to retroflex in the rectum?	Feel rushed?	Prolong a procedure to meet quality standards for withdrawal time?	Remove diminutive polyps solely to increase your adenoma detection rate?
Sex	*P*	0.260	**0.048**	0.185	0.744	0.978	0.397
	*η* ^2∗∗^	0.009	**0.025**	0.012	0.001	0.000	0.005

Years in practice	*P*	0.701	0.593	0.446	0.456	0.152	0.244
	*η* ^2^	0.001	0.002	0.004	0.004	0.015	0.009

GI board certification	*P*	0.382	0.231	0.725	0.835	0.258	0.121
	*η* ^2^	0.005	0.009	0.001	0.000	0.009	0.016

Practice setting	*P*	0.911	**0.008**	0.808	**0.018**	0.413	0.406
	*η* ^2^	0.000	**0.046**	0.000	**0.040**	0.005	0.005

Productivity bonus	*P*	0.603	0.260	0.914	0.621	0.503	0.159
	*η* ^2^	0.002	0.008	0.000	0.002	0.003	0.013

Colonoscopy volume	*P*	0.695	0.610	0.647	0.810	0.543	0.069
	*η* ^2^	0.016	0.017	0.017	0.011	0.020	0.058

Receive quality feedback	*P*	0.647	**0.004**	0.739	0.554	0.824	0.843
	*η* ^2^	0.002	**0.053**	0.001	0.002	0.000	0.000

*Response categories include never, rarely, sometimes, usually, and always.

**Eta squared (*η* ^2^) represents the proportion of variance in the dependent variable explained by the independent variable.

**Table 5:** Physician perceptions about colonoscopy quality benchmarks.

		With regard to the guideline recommendation of an average withdrawal time of 6 minutes for a colonoscopy without polyps, do you feel this is…?*	With regard to the guideline recommendation of an average adenoma detection rate of 15% for women and 25% for men, do you feel this is…?**
Sex	*P*	0.592	0.811
	*η* ^2∗∗∗^	0.002	0.000

Years in practice	*P*	0.320	**0.001**
	*η* ^2^	0.007	**0.078**

GI board certification	*P*	0.786	0.619
	*η* ^2^	0.001	0.002

Practice setting	*P*	0.989	0.338
	*η* ^2^	0.000	0.006

Productivity bonus	*P*	0.165	0.920
	*η* ^2^	0.013	0.000

Colonoscopy volume	*P*	0.069	0.664
	*η* ^2^	0.059	0.015

Receive quality feedback	*P*	0.094	**0.003**
	*η* ^2^	0.019	**0.058**

*Response categories include too short, about right, and too long.

**Response categories include too low, about right, and too high.

***Eta squared (*η* ^2^) represents the proportion of variance in the dependent variable explained by the independent variable.

**Table 6:** Physician perceptions about colonoscopy surveillance recommendations*.

		Patient preference	Adequacy of the preparation	Difficulty of the procedure	Financial incentives	Malpractice concerns
Sex	*P*	0.658	**0.007**	0.821	0.262	0.128
	*η* ^2∗∗^	0.001	**0.057**	0.000	0.009	0.015

Years in practice	*P*	0.401	0.741	0.091	0.625	**0.001**
	*η* ^2^	0.005	0.001	0.022	0.002	**0.073**

GI board certification	*P*	0.981	0.549	0.224	**0.036**	0.691
	*η* ^2^	0.000	0.003	0.011	**0.032**	0.001

Practice setting	*P*	**0.004**	0.328	0.489	0.110	**0.002**
	*η* ^2^	**0.062**	0.007	0.004	0.018	**0.067**

Productivity bonus	*P*	0.343	0.690	0.852	0.372	0.148
	*η* ^2^	0.007	0.001	0.000	0.006	0.014

Colonoscopy volume	*P*	0.991	0.859	0.751	0.260	**0.038**
	*η* ^2^	0.002	0.010	0.015	0.037	**0.069**

Receive quality feedback	*P*	0.415	0.728	0.490	0.100	0.185
	*η* ^2^	0.005	0.001	0.004	0.019	0.012

*The root of each question was as follows. “Rate how often you shorten a surveillance recommendation based on…never, rarely, sometimes, usually or always?”

**Eta squared (*η* ^2^) represents the proportion of the variance in the dependent variable that can be explained by the variance in the independent variable.
